# Cancer-associated fibroblasts in gastric cancer affect malignant progression via the CXCL12-CXCR4 axis

**DOI:** 10.7150/jca.49707

**Published:** 2021-03-19

**Authors:** Yan Qin, Fang Wang, Hengli Ni, Yao Liu, Yuan Yin, Xinyi Zhou, Guihua Gao, Qing Li, Xiaowei Qi, Jianming Li

**Affiliations:** 1 Department of Pathology, Medical College of Soochow University, Soochow University, Suzhou, China.; 2 Department of Pathology, the Affiliated Hospital of Jiangnan University, Wuxi, China.; 3 Jiangnan University School of Medicine, Wuxi, China.; 4 Wuxi Oncology Institute, the Affiliated Hospital of Jiangnan University, Wuxi China.; 5 Department of Oncology, The Second Affiliated Hospital of Soochow University Suzhou, China.

**Keywords:** gastric cancer, microenvironment, CAFs, scRNA, CXCL12

## Abstract

**Background:** Cancer-associated fibroblasts (CAFs) are principal constituents of the tumor microenvironment (TME) and play a critical role in tumor progression. The CXCL12/CXCR4 axis regulates multiple facets of the TME. The aim of this study was to determine the relationship between CXCL12 expression in CAFs and the malignant progression of gastric cancer (GC).

**Methods:** In the GEO (Gene Expression Omnibus) database, we performed transcriptome analysis on paired gastric cancer RNA sequencing samples, and scRNA analysis was performed on advanced malignant GC samples from the scRNA sequencing data set. Fibroblast cells were co-cultured with GC cells, and invasion, migration, epithelial-mesenchymal transformation (EMT) were determined. After blocking the expression of fibroblast CXCL12, cells were co-cultured with a GC cell line. Detection of GC cell line invasion, migration, EMT and CXCR4, Wnt5a and β-Catenin expression levels was performed. Primary CAFs and gastric normal fibroblasts were isolated and CXCL12 mRNA and protein expression were determined. In addition, a cohort of 285 GC cases was established, protein expression was evaluated immunohistochemically, and prognostic results were analyzed.

**Results:** GC transcriptome analysis suggested that cytokine-cytokine receptor interaction and the Wnt signaling pathway in GC tissues were significantly up-regulated. scRNA analysis of advanced malignant GC samples showed that severe intestinal metaplasia (SIM) in GC specimens of different malignant grades had obvious fibroblast clusters compared to non-atrophic gastritis (NAG) and early gastric cancer (EGC). In the SIM group, fibroblast cluster, CXCL12, CXCR4, and Wnt5a were overexpressed. Co-culturing with fibroblast cells significantly increased the invasion, migration, and EMT of GC cells, and blocking CXCL12 in CAFs disturbed the expression of Wnt5a and β-catenin. In our cohort of GC patients, high CXCL12 expression in CAFs significantly correlated with histological grade (P = 0.012) and TNM stage (P = 0.014), as well as with poor overall survival (p = 0.0107).

**Conclusion:** High expression of CXCL12 in CAFs in a GC microenvironment can affect the migration, invasion, and EMT of GC cells. Furthermore, it can cause poor prognosis in patients with GC.

## Introduction

Gastric cancer (GC) is a commonly diagnosed lethal malignancy that is highly prevalent worldwide 1,2. Infiltration and metastasis of GC cells are the main causes of poor prognosis and mortality among GC patients 3. The critical role of the tumor microenvironment (TME) in the growth and metastasis of malignant cells is well known 4. Thus, this is an important research direction of the malignant progression mechanism and clinical treatment of GC.

CXCL12 (CXC ligand 12, stromal cell-derived factor-1, SDF-1) is a chemotactic cytokine or chemokine that functions as a signaling protein. Recent studies have shown that CXCL12 is produced by stromal cells, secreted in the TME, and binds to CXCR4 on the surface of tumor cells, which is crucial for tumor metastasis and poor prognosis 5-7. However, the biological role of CXCL12 in GC has yet to be evaluated using multi-method integration. In addition, the prognostic relevance of EMT and the role of the Wnt5a/β-catenin axis in GC remain elusive 8.

In this study, we performed integrated analysis of the RNA seq GC tissues and normal tissues, and evaluate CAFs infiltrating cancer tissues and normal tissues. Moreover, scRNA sequencing analysis of GC tissues with different malignant grades was performed. In addition, the expression levels of CXCL12 in GC-derived CAFs was analyzed, and correlated with β-Catenin and Wnt5a 9. Finally, the clinical significance of Wnt5a and CXCL12-secreting CAFs was determined. Taken together, our findings provide novel insights into the mechanisms underlying GC progression and potential targeted therapeutic strategies.

## Methods

### Integrated bioinformatics analysis

The mRNA sequencing datasets of GC were searched from the NCBI-GEO database based on the following inclusion criteria: a) data from both tumor and normal tissue 10, b) Illumina HiSeq 2000 or a later version, and c) data retrieved after 2015. Next, the original SRA (Sequence Read Archive) file and the expression matrix file of the published datasets were downloaded and validated. The exclusion criteria were as follows: 1) duplicate reports uploaded by the same research institution, 2) lack of human genome-wide files 11, and 3) original file was transferred to the fastq files, compared to the human reference genome hg38, and the matching rate was less than 90%. Normalization of each dataset was performed on the base-2 logarithm by the Robust Multi-Array Average (RMA) and Linear Models for Microarray (LIMMA) package 12. mRNA was converted to gene id format, Gene Ontology (GO) and Kyoto Encyclopedia of Genes and Genomes (KEGG) analysis based on clusterProfiler 13. Gene set enrichment analysis (GSEA) analysis was also performed using enrichplot and ggplot2 packages. EPIC software (GfellerLab/EPIC, https://gfellerlab.shinyapps.io/EPIC_1-1/) was used to infiltrate fibroblasts from GC and normal tissues 14. The scRNA sequencing datasets of GC were searched through the NCBI-GEO database and were based on a 10×Genomic or SMART-Seq2 platform. The scRNA sequencing analysis progress used Seurat, SingleR and magrittr packages were used for the dimensionality reduction analysis in UMAP and TSNE methods. Trajectory analyzed that we used the Monocle package. All data analyzed were included in the GSE122796 and GSE134520 datasets 15. External independent samples were used to verify the survival of patients, and the TIMER tool (Tumor Immunity Estimation Resource, https://cistrome.shinyapps.io/timer/) was used to analyze the survival of the gastric adenocarcinoma dataset (STAD) of the TCGA database using Kaplan-Meier survival analysis 16.

### Cell culture and treatment

Human GC cell lines SGC7901 and BGC823, and human fibroblast cell line MRC-5 were obtained from American Type Culture Collection (ATCC, Manassas, VA, USA). GC cell lines were cultured in Dulbecco's modified eagle's medium (DMEM)/F12 (Gibco, New York, USA), and MRC-5 in minimum essential medium (MEM) (Hyclone, Utah, USA), supplemented with 10% fetal bovine serum (FBS, Gibco, USA) and 1% streptomycin (100 U/mL) -penicillin (100 U/mL) (Beyotime, Nantong, China), in a humidified atmosphere of 5% CO_2_ at 37℃. MRC-5 cells were treated with the CXCL12 blockers CS-1448 (SC1166, Beyotime, Nantong, China) and Plerixafor (SD2380, Beyotime, Nantong, China). For CXCL12 silencing, cells were plated in 6-well plates at a density of 5×10^5^ cells per mL, washed once with PBS, and transfected with 100 nM CXCL12 si-RNA or negative control (NC) (Gene Pharma, Shanghai, China) using Lipofectamine 3000 (Invitrogen, New York, USA) according to the manufacturer's instructions.

### Cell migration and invasion

Cell migration and invasion assays were performed using 8 μm transwell inserts in 24-well plates (Corning Life Science, Bedford, MA, USA). To evaluate migration, tumor cells were seeded into the upper chamber at a density of 5×10^4^ cells/200 µL serum-free medium, and 2×10^4^ MRC-5 were plated in the lower chamber in 800 μL complete medium. For the invasion assay, the transwell membranes were coated with 50 μL Matrigel per well (BD Bioscience, CA, USA) before cells were added. Cells were cultured for 24 h and stained with 0.1% crystal violet for 30 min after removal of non-migrating/invading cells. Migrated/invaded cells were counted in six randomly selected fields.

### Western blot analysis

Cells and tissues were homogenized for 25 min on ice in RIPA lysis buffer, supplemented with protease inhibitors, phosphatase inhibitors, and 100 mM PMSF (KeyGEN BioTECH, Shanghai, China). Extracted proteins were resolved by 10% SDS-PAGE and transferred to polyvinylidene fluoride (PVDF) membranes (Merck Millipore, Darmstadt, Germany) using a semi-dry apparatus. Membranes were blocked in 5% skimmed milk in Tris-buffered saline containing 0.1% Tween-20 (TBST) for 1h at room temperature, followed by overnight incubation with antibodies targeting CXCL12 (1:1000, 3530, Cell Signaling Technology), CXCR4 (1:1000, 11073-1-AP, Proteintech), Wnt5a (1:1000, ab174963, Abcam), β-Catenin (1:5000, ab174963, Abcam), E-cadherin (1:5000, AF1552, Beyotime), N-cadherin (1:2000, AF0243, Beyotime), Vimentin(1:5000, AF0218, Beyotime) and GAPDH (1:2000, ab181602, Abcam) at 4 °C. After washing three times with TBST for 10 min at room temperature, membranes were incubated with secondary antibodies for 1 h, and washed three times with TBST. Positive bands were visualized using an ECL Plus kit (Beyotime, China).

### Quantitative RT-PCR assays

Total RNA was extracted from the cells and tissues using Trizol reagent (Invitrogen, Carlsbad, CA, USA). HiScript II 1^st^ Strand cDNA Synthesis Kit (Vazyme, Nanjing, China) was used for cDNA synthesis according to the manufacturer's guidelines. Gene expression levels were determined using a QuantiNovaTM SYBRR Green PCR Kit (Qiagen, Hilden, Germany) with β-actin as the internal control. The primer sequences were as follows: β-actin: F: CTCCATCCTGGCCTCGCTGT, R: GCTGTCACCTTCACCGTTCC; CXCL12: F: GCCATGAACGCCAAGGTC, R: CGAGTGGGTCTAGCGGAAAG; Wnt5a: F: CTTCAACTCGCCCACCACACAAGA, R: CACACAAACTGGTCCACGATCTCC. The amplification conditions were as follows: denaturation at 95 °C for 5 min, followed by 35 cycles at 95 °C for 10 s, and 60 °C for 30 s.

### Isolation of primary fibroblasts

GC tissues were obtained from patients undergoing surgery at the Affiliated Hospital of Jiangnan University (Wuxi, China). Tissues were minced (1 to 2 mm pieces) and digested with DMEM/F12 (Gibco, New York, USA) containing 10% FBS, 2 mg/mL collagenase I and 2 mg/mL hyaluronidase (Beyotime, Nantong, China) at 37 °C for 2 h. The homogenate was centrifuged at 3000 rpm for 15 min in 437 °C, and the resulting adherent cells were digested again. CD271-positive CAFs were isolated using the CD271 MicroBead Kit (APC, Mainz, Germany) according to the manufacturer's instructions, and cultured in DMEM/F12 medium until 100% confluent.

### Patient samples and microarray (TMA) construction

Tissue samples were resected from 285 GC patients at the Affiliated Hospital of Jiangnan University between 2008 and 2014. The original histological sections were reviewed independently by two researchers. Patients clinicopathological parameters, including gender, age, tumor size, histological grade, primary tumors (pTs), nodal (pN) metastasis, pathological stage, vascular invasion, neural invasion, and lymphatic invasion, were analyzed. The mean follow-up duration was 21.3 months, and ranged from 3-89 months. Our study was approved by the Ethics Review Board of the Affiliated Hospital of Jiangnan University (Wuxi, China).

Tissue microarrays (TMA) were assembled using artificial tissue microarray spotting (Quick-Ray; UNITMA Co., Ltd, Seoul, Korea). Two representative tumor and adjacent fibroblast cores measuring 1.5 mm in diameter were obtained from the same tissue block of each patient, and histologically verified by Hematoxylin and eosin (H&E) staining. Cases with inadequate carcinoma tissue or lack of carcinoma or fibroblasts tissue in the cores were not included.

### Immunohistochemical detection of pathological specimens

A Leica paraffin slicer RM2235 (Leica Biosystems, Solms, Germany) was used to cut 4 µm-thick sections from each TMA block. Sections were incubated at 70 °C for 1 h, dewaxed in xylene, and rehydrated using an ethanol gradient. Following antigen retrieval by boiling in citrate buffer for 20 min, sections were incubated in hydrogen peroxide for 10 min to quench endogenous peroxidase. Then, tissues were incubated overnight with primary polyclonal antibodies targeting CXCL12 (1:100, 3530, Cell Signaling Technology, Manassas, USA), Wnt5a (1:200, ab174963, Abcam) and β-Catenin (1:300, ab230216) at 4 °C. PBS was used as a negative control. After washing with PBS, sections were incubated with binding buffer and amplification agent (reagent A, GTVisionTM III Kit supply, Shanghai, China), stained with 3, 3'-diaminobenzidine (DAB, reagent B and C, GTVisionTM III Kit supply, Shanghai, China), and counterstained with hematoxylin. Staining was scored based on the percentage of positively-stained cells in each section (no positive staining or ≤ 5% = 0; 6%-25% = 1; 26%-50% = 2; 51%-75% = 3, and 76%-100% = 4), and the staining intensity (no staining = 0, weak staining = 1, moderate staining = 2 and strong staining = 3) 17. For each case, two random fields were imaged, with one core viewed under high magnification (×200). All slides were analyzed by two pathologists in a blinded manner.

### Statistical analysis

Appropriate cutoff values for CXCL12, Wnt5a, and β-catenin expression were obtained from the receiver operating characteristic curve (ROC) and area under curve (AUC). Pearson's Chi-square test or Fisher's exact test was used to determine the association between CXCL12, Wnt5a and β-catenin expression, and the clinicopathological parameters. Overall survival (OS) was plotted by the Kaplan-Meier method and analyzed using the log-rank test. Univariate and multivariate survival analyses were performed using the Cox proportional hazard model. Univariate and multivariate survival analyses were performed by the Cox regression model, and a ROC curve was plotted to evaluate the predictive power of the different factors on patient prognosis. P<0.05 was considered statistically significant.

## Results

### Identification of GC transcriptome analysis and CAFs in microenvironment

**Figure 1:**
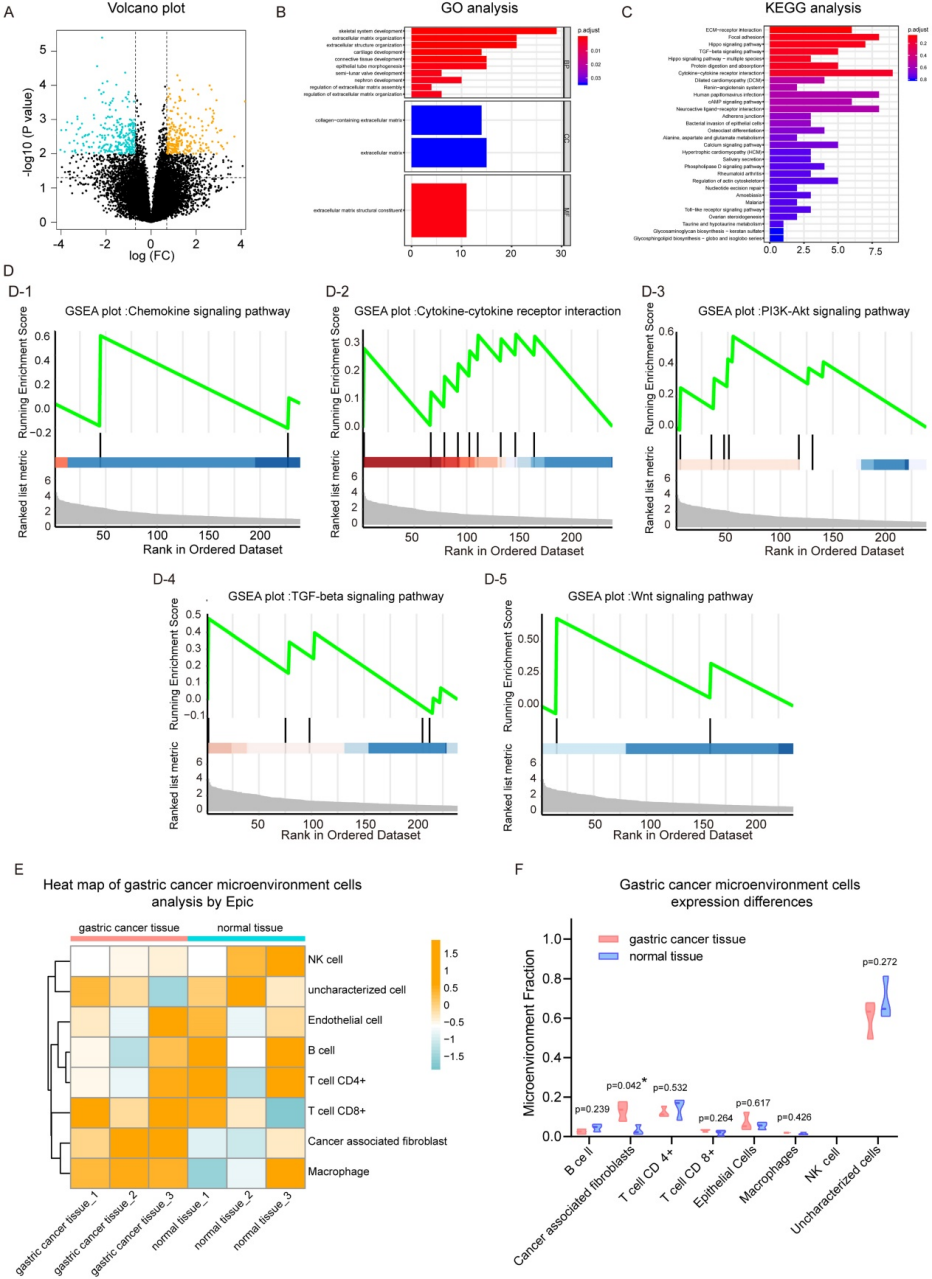
** Analysis of gastric cancer and paracancer transcriptome data set.** (A) Volcano plot of gene expression. (B) Gene ontology (GO) analysis plot of up-regulated genes in gastric cancer (GC) tissues. (C) Kyoto Encyclopedia of Genes and Genomes (KEGG) analysis of up-regulated genes in GC tissues. (D) Gene set enrichment analysis (GSEA) plot based on the KEGG signaling pathway; D-1, chemokine signaling pathway; D-2, cytokine-cytokine signaling pathway; D-3, PI3K-Akt signaling pathway; D-4, TGF-beta signaling pathway; D-5, Wnt signaling pathway. (E) EPIC heat map analysis of cell types of GC tissues and adjacent normal tissues. (F) Violin plots with different expression of various cell types adjacent to cancer tissue.

We first analyzed the mRNA sequencing data sets of three pairs of GC tissue and adjacent normal tissues from GSE122796. After normalizing and merging of the same genes, a total of 18793 genes were analyzed. After cut-off value analysis (P<0.01, fold change >2), 239 genes were found to be up-regulated in GC tissues and 269 genes were down-regulated in GC tissues (Figure 1A, and heat map Figure S1B). Next, functional enrichment analysis was performed on all 239 genes that were up-regulated in cancer tissues. Our data showed that in the GO analysis, extracellular matrix organization, extracellular structure organization, and epithelial tube morphogenesis were up-regulated. The molecular function of extracellular matrix structural constituents was enhanced, In Figure 1B. In the results of KEGG analysis, we found that Cytokine-cytokine Receptor interaction pathway was significantly up-regulated. In addition, the Hippo signaling pathway, TGF-beta signaling pathway, and cAMP signaling pathway as well as other tumor-related signaling pathways were significantly activated.

**Figure 2:**
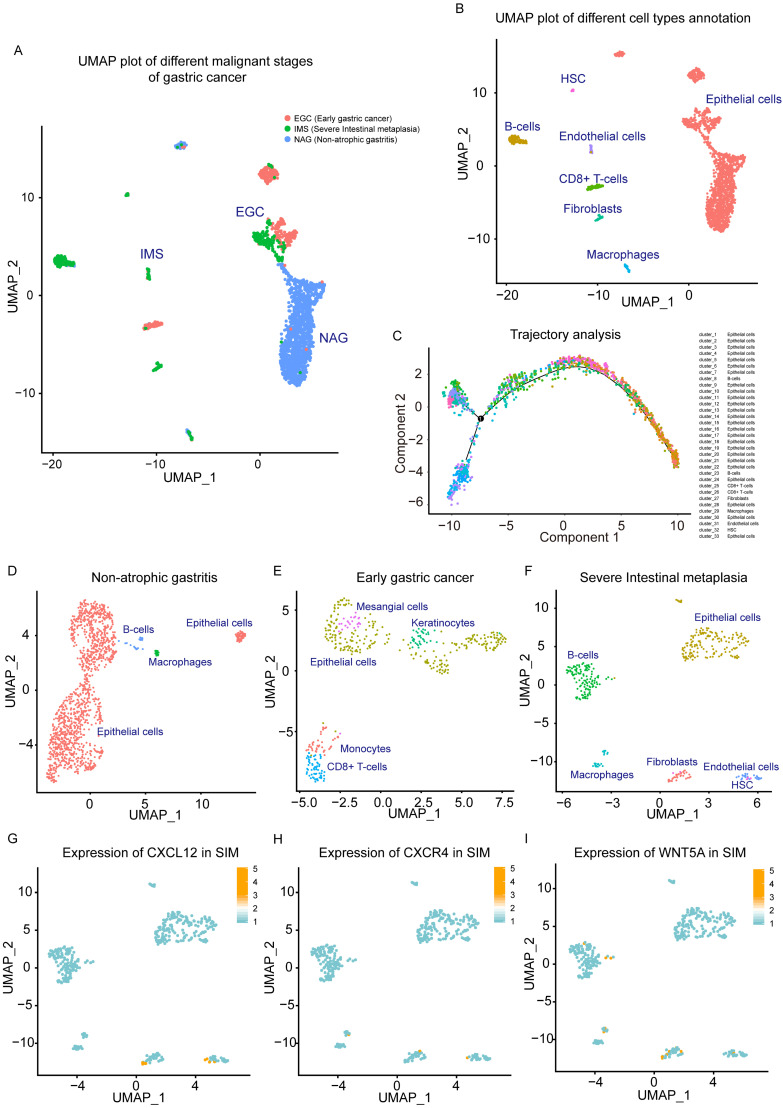
** Single-cell analysis of non-atrophic gastritis (NAG), early gastric cancer (EGC) and severe intestinal metaplasia (IMS) samples.** (A) UMAP plot of NAG, EGC, and IMS single-cell samples. (B) NAG, EGC, and IMS single-cell samples. Cluster markers of different infiltrating cell types (C) trajectory analysis plots of different cell types. (D) UMAP plot of cell type distribution in the NAG group. (E) UMAP plot of cell type distribution in the EGC group. (F) UMAP plot of cell type distribution in the SIM group. (G) Expression of CXCL12 in SIM group. (H) CXCR4 expression in the SIM group. (I) Expression of Wnt5a in the SIM group.

**Figure 3:**
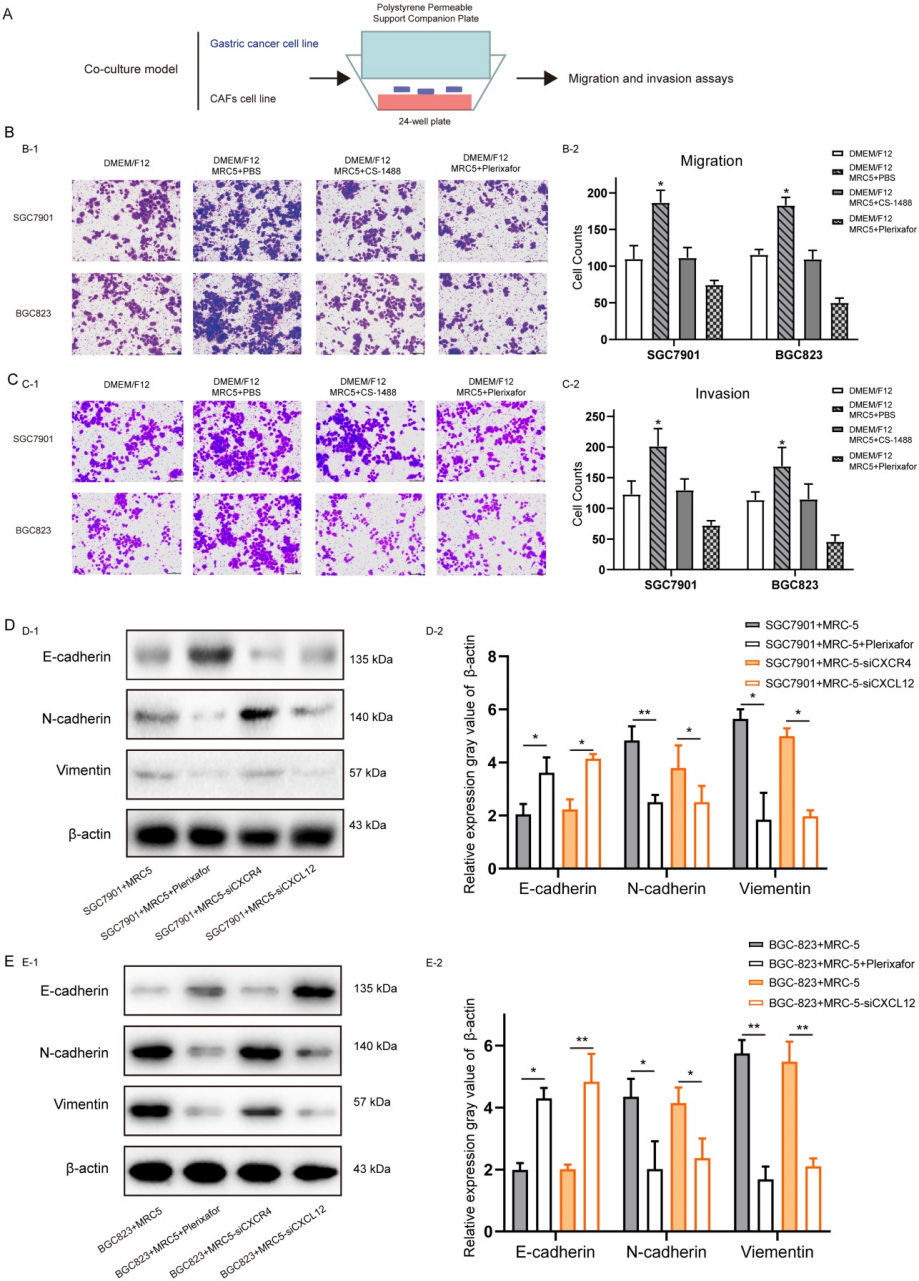
** The effect of CXC12 expression in Cancer-associated fibroblasts on the migration, invasion and Epithelial-mesenchymal transition of gastric cancer cells.** (A) Schematic diagram of co-culture of fibroblasts (MRC-5) and gastric cancer (GC) cells (SGC7901 and BGC823). (B) Number of migrating GC cells co-cultured with control and CXCL12-blocked MRC-5 cells. (C) Number of invading GC cells co-cultured with control and CXCL12-blocked MRC-5 cells. (D) After co-culture of SGC7901 cells with MRC-5, MRC-5 + Plerixafor, MRC-5 interfered with CXCR4 and MRC-5 interfered with CXCL12, the expression levels of E-cadherin, N-cadherin and Vimentin. (E) After co-culture of the BGC823 cell line with MRC-5, MRC-5 + Plerixafor, MRC-5 interfered with CXCR4 and MRC-5 interfered with CXCL12, the expression levels of E-cadherin, N-cadhrin and Vimentin.

The 239 genes that were significantly up-regulated in GC were combined with their expression levels relative to adjacent normal tissues, and gene set enrichment analysis was performed. Results for the KEGG pathway gene set enrichment are shown in Fig. 1d. In cancer tissues, chemokine (Figure 1D-1), cytokine-cytokine (Figue 1D-2) and other tumor progress-related pathways were up-regulated in GC tissue. Regarding KEGG plot analysis results, PI3k-Akt and TGF-β signaling pathways were up-regulated (Figure 1D-3, 1D-4). We also found that the Wnt signaling pathway was activated in cancer tissues, which was consistent with the results of our previous study. In addition, we performed gene set enrichment analysis based on GO (Figure S1C), which was consistent with the aforementioned results.

Subsequently, EPIC software was used to analyze the infiltrating cell types of these three pairs of cancer and adjacent normal tissues. The overall cell type profile is shown in Figure 1E and Figure S1-D. We found that the cancer-associated fibroblast fraction in cancer tissue was significantly higher when compared to that in adjacent tissue (Figure 1F; P<0.05). Our data showed that there were more fibroblasts present in cancer tissues compared to adjacent tissues. Furthemore, multiple signaling pathways, including cytokine-cytokine, chemokine, PI3k-Akt, and Wnt were activated in cancer tissues.

### Single-cell sequencing analysis found CAFs are associated with malignant progression of GC

To further study the cell types of the tissue microenvironment of GC tissue, and the effect of microenvironment infiltrating cells on tumor progression, single-cell transcriptome analysis was performed based on the GEO dataset GSE134520. From this data set, 3 types of single-cell sequencing samples were selected with different progress of gastric tissue as NAG, EGC and IMS. We reduced the dimension and used UMAP analysis of the 3 source types of single-cell data (Figure 2A). In single-cell sequencing samples of GC tissue, we found large differences between these 3 categories. NAG-derived cells have different distribution characteristics from EGC and IMS, which was also reflected by TSNE analysis (Figure S2-E). We performed cell type labeling analysis on single cell data sets of NAG, EGC, and IMS. We found that Fibroblasts Cluster in GC tissues and Fibroblasts Cluster only appeared in the IMS group (Figure 2B). These findings showed that CAFs represent an identified proportion in malignant GC tissues. Through trajectory analysis, the study of the Trajectory tracking of cell types in GC scRNA samples also reflected the results, as shown in Figure 2C.

Single-cell data sets of EGC, SIM, and NAG were analyzed. After single-cell cluster analysis, we found that NAG mainly contained epithelial cells, and a small amount of B cells and macrophages (Figure 2D). The EGC was more complicated, and showed infiltration of CD8 + T cells and monocytes (Figure 2E). In the SIM group, with more severe malignant GC, there were more prominent fibroblast clusters. In addition, clusters of B cells, macrophages, and endothelial cells were clearly clustered in SIM (Figure 2F). Based on the results of our functional enrichment analysis of the GC transcriptome and our previous study, we analyzed the expression of cytokine-cytokine signaling molecules CXCL12 and CXCR4 in SIM GC samples. CXCL12 was mainly expressed in fibroblasts and endothelial cells (Figure 2G), and CXCR4 was mainly expressed in clusters of CAFs and epithelial cells (GC cells included) (Figure 2H). In addition, Wnt5a in fibroblasts cluster was high expression (Figure 2I).

### CAFs promote migration, invasion, and EMT of GC cells by CXCL12 overexpression

The results of integrated bioinformatics analysis suggested that the malignant progress of GC was accompanied by cytokine-cytokine signaling, and the activation of Wnt signaling pathway may be closely related to CAFs in GC tissues.

The role of CXCL12 expression in CAFs (CXCL12^CAF^) induce GC cell migration, and invasion was analyzed using an *in vitro* indirect co-culture system (Figure 3A). Transwell assays showed that blocking the secretion of CXCL12 by CS-1448 and Plerixafor from MRC5 cells significantly decreased the migration and invasion of GC cells compared to MRC5 cells co-cultured with untreated CXCL12 in fibroblasts (Figure 3B and Figure 3C). Furthermore, SGC7901 cells co-cultured with CAFs for 24 hours expressed markedly lower levels of the epithelial marker E-cadherin and high levels of the mesenchymal N-cadherin and Vimentin (Figure 3D), indicating that CXCL12 secretion by fibroblasts enhanced the EMT of GC cells. Moreover, when we were blocked with Plerixafor on MRC-5 in CXCL12, or knocking down the expression of CXCL12, GC cell migration, invasion, and EMT were suppressed (Figure 3D). Similar results were obtained when using BGC823 cells (Figure 3E). Taken together, activated CAFs promote GC cell migration, invasion, and EMT via CXCL12.

### Correlation between CXCL12 and Wnt5a in GC cells

**Figure 4:**
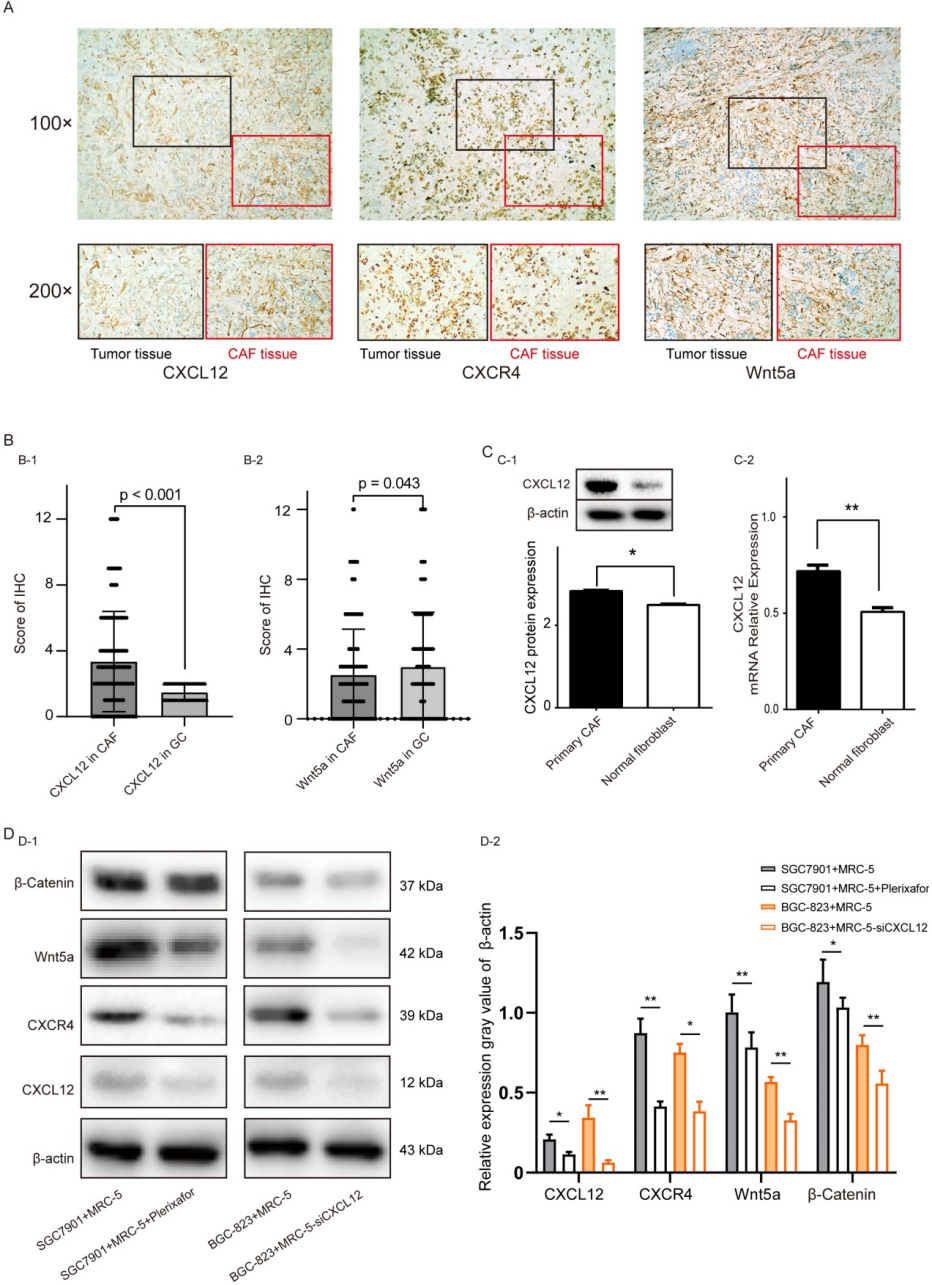
** Cancer-associated fibroblasts microenvironment of CXCL12 mechanism of action of gastric cancer cells.** (A) Representative images of gastric cancer (GC) and CAF tissue microarrays showing HE-staining and IHC analysis of CXCL12 and Wnt5a (50× and 200× magnification). (B) IHC scores of CXCL12 and Wnt5a in GC tissues and CAFs, P<0.05. (C) CXCL12 protein (C-1) and mRNA (C-2) levels following knockdown in primary CAFs and NFs. (D) CXCL12, CXCR4, Wnt5a and β-Catenin levels in SGC7901 cells co-cultured with MRC-5 and MRC-5+Plerixafor, and in BGC823 cells co-cultured with MRC-5 and MRC-5-siCXCL12 cells.

We used immunohistochemistry experiments on GC tissues to locate the expression of CXCL12 and Wnt5a protein. CXCL12 and Wnt5a were expressed in the cytoplasm of tumor-adjacent fibroblasts and the GC cells, respectively (Figure 4A). In addition, CXCL12 expression was significantly higher in CAFs when compared to GC cells (P<0.001), while Wnt5a expression was higher expression in GC cells (P<0.043, Figure 4B). Consistent with this, CXCL12 was significantly up-regulated in primary CAFs compared to primary normal fibroblasts, both at the protein level (P<0.05, Figure 4C-1) and mRNA level (P<0.01, Figure 4C-2). We further verified the relationship between CAFs and the expression of Wnt5a in GC cells in cell lines. After co-culturing of SGC7901 with MRC-5 cells and treatment of MRC-5 cells with Plerixafor, respectively, we found that the expression levels of CXCL12, CXCR4, Wnt5a, and β-Catenin protein in SGC7901 cells was decreased in co-cultures of MRC-5 cells treated with Plerixafor. In co-culture experiments with BGC823 cells, the expression level of mRNA CXLC12 was knocked down in a co-culture of MRC-5 cells, we also found that the CXCL12, CXCR4, Wnt5a, and β-Catenin protein level was decreased in a co-culture with MRC-5 treated with siCXCL12 (Figure 4D-1 and Figure 4D-1). We also used TCGA-STAD GC tissue samples to predict the expression levels of CXCL12 and fibroblast markers (Figure S3). We found that CXCL12 is positively related with Vimentin (P<0.0001), α-actin (P<0.0001), and HSP47 (P<0.0277).

### Correlation between CXCL12^CAF^, Wnt5a expression, and clinicopathological features

**Figure 5:**
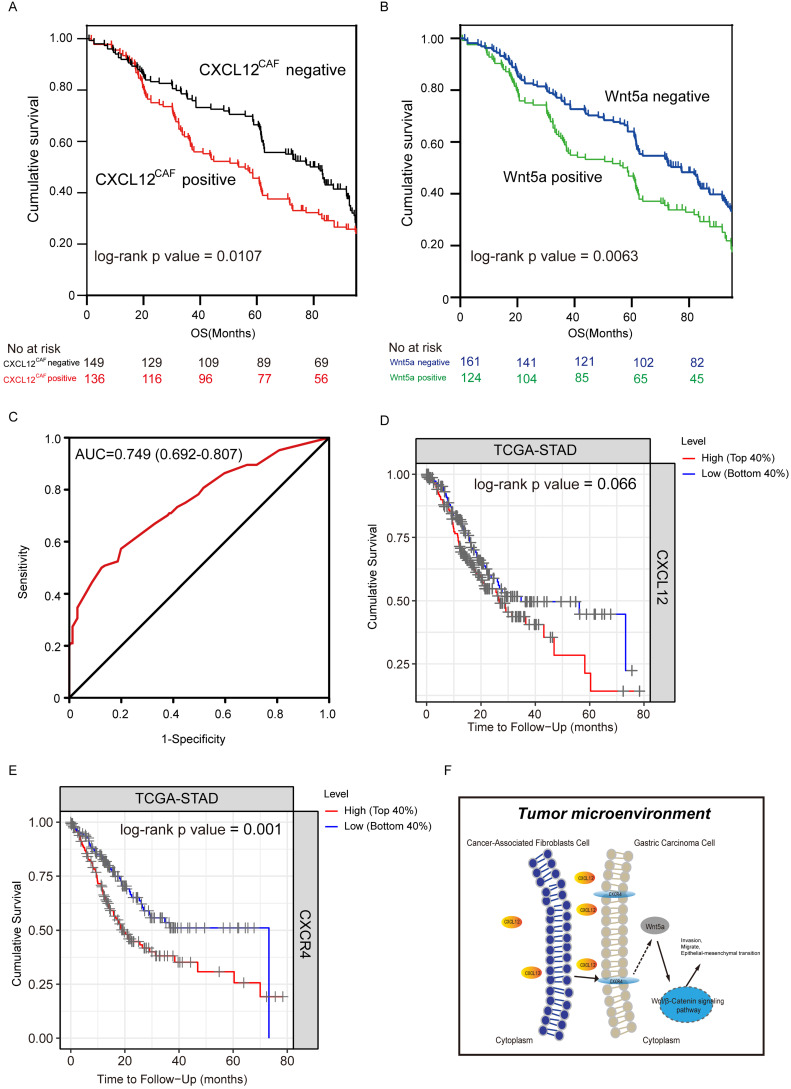
** Effect of high expression of CXCL12 in Cancer-associated fibroblasts on survival of gastric cancer patients.** Kaplan-Meier curves showing the impact of CXCL12^CAF^ and overall survival (OS) in 285 gastric cancer (GC) patients, P=0.0107. (B) Kaplan-Meier curves showing the impact of Wnt5a on the OS of patients, P=0.0063. (C) ROC curve showing the performance of the multivariate regression prediction model on patient prognosis, AUC = 0.749. (D) Kaplan-Meier curves showing the impact of CXCL12 on the OS of patients in TCGA-STAD dataset by TIMER tools, P = 0.066. (E) Kaplan-Meier curves showing the impact of CXCR4 on the OS of patients in the TCGA-STAD dataset by TIMER tools, P = 0.001. (F) Schematic diagram of CAFs affecting the progression of GC cells by secreting CXCL12.

In our cohort, clinicopathological and demographic features of 285 GC patients are shown in Table 1. CXCL12 was predominantly expressed in the cytoplasm of fibroblasts adjacent to GC cells, while Wnt5a was localized in the cytoplasm of GC cells (Figure 5A). As shown in Table 1, high CXCL12 expression in CAFs significantly correlated with the histological grade (P=0.012) and TNM stage (P=0.014), while Wnt5a expression was associated significantly with primary tumor category (P=0.016). Based on the IHC score of Wnt5a and CXCL12 expression in the GC area and adjacent fibroblast area, CXCL12^CAF^ expression was significantly higher than CXCL12 in gastric adenocarcinoma (P<0.001). Thus, these findings suggested that the effect of CXCL12 secreted by CAFs on GC cells was stronger. Wnt5a expression in CAFs (Wnt5a^CAF^) was significantly lower than Wnt5a in gastric adenocarcinoma (P<0.05) (Figure 5B). As shown in Table 2, univariate analysis showed that the tumor size (HR = 1.29, 95% CI= 1.00-1.66, P=0.047), primary tumor category (HR = 0.55, 95% CI= 0.43-0.70, P<0.001), Lauren type (HR = 1.58, 95% CI= 1.15-2.23, P=0.001), TNM stage (HR = 1.13, 95%CI= 1.02-1.34, p = 0.046), CXCL12^CAF^ (HR = 1.38, 95%CI= 1.07-1.76, P=0.012) and Wnt5a (HR = 1.10, 95%CI= 1.41-1.81, P=0.007) significantly correlated with OS. Furthermore, multivariate Cox regression analysis indicated that primary tumor category (HR = 0.55, 95% CI= 0.42-0.72, P<0.001), Lauren type (HR = 1.64, 95% CI= 1.23-2.17, P=0.001) and CXCL12^CAF^ were related with a relative risk of death of 1.50 (95% CI, 1.16-1.93, P=0.002). The CXCL12^CAF^ high expression patients (n = 136) showed significantly worse survival than the CXCL12^CAF^ negative subgroup (n = 149) (log-rank P=0.017). Consistent with this, patients with high expression Wnt5a (n=124) had significantly lower cumulative survival rates compared to patients with low Wnt5a expression (n=161) (log-rank P=0.0063, Figure 5B). We next combined CXCL12^CAF^ with primary tumor category and Lauren type to establish a logistic regression model to predict the five-year survival of GC patients. As shown in the ROC plot in Figure 5C, the area under the curve (AUC) was 0.749, and the P-value was 0.024, suggesting a high predictive value of the diagnostic model for the five-year survival of GC patients. In addition, to further verify the results of the study, we used the TIMER tool and employed Kaplan-Meier survival analysis on the CXCL12 and CXCR4 groups of patients in TCGA-STAD. We found that the survival of patients in the CXCL12 mRNA high expression group was worse than patients in the CXCL12 low expression group, Log-rank P=0.066 (Figure 5D). The survival rate of the CXCR mRNA high expression group was significantly lower than that of the CXCR4 mRNA low expression group, log-rank P=0.001 (Figure 5E).

## Discussion

CXCL12, also known as stromal cell derived factor SDF-1, is a chemokine secreted by multiple cell types that participate in various physiological and pathological processes 18-23. CXCL12 binds to metastasis-related proteins in GC cells 24, and is therefore a biomarker of tumor prognosis, metastasis, and chemo-resistance 25. However, CXCL12 fails to distinguish between fat and lean mass, and exerts differential effects on the regulation of the immune microenvironment and GC survival 26. In recent years, Wnt5a signaling in CAFs has been implicated in tumor progression 27. Overexpression of β-Catenin and Wnt5a promotes cell growth, migration, invasion, and EMT of digestive tract tumors 28, and are thus indicators of poor prognosis.

It was recently demonstrated that targeting both cancer cells and TME angiogenesis could decrease tumor growth and metastasis in CXCL12^CAF^ of GC patients 29. For this reason, both are practical and effective treatment options to target tumor cells, CAFs, and the TME. The latest treatment strategies aim to eliminate CAFs, or restore the activation status of CAFs to a resting state. However, challenges remain and improvements in this area are warranted. This study provides insight into the selection of candidate targets for combination therapies that target the crosstalk between gastric carcinoma cells and CAFs. We found that CXCL12 in CAFs significantly associated with high expression and poor prognosis in obese patients. In addition, sequencing data from GEO gastric adenocarcinoma CAFs, and comprehensive analysis of the transcriptome sequencing of our collection of specimens, greatly reduced the false positives of single-chip analysis, thereby providing a more reliable basis for the follow-up research. Moreover, survival analysis of CXCL12 and CXCR4 mRNA levels combined with the TCGA-STAD data set verified these results. It is worth noting that although the log-rank P value of CXCL12 was 0.066, it was considered that the TCGA data set was a typical bulk-sequencing data set, and as our study emphasized, CXCL12 had more influence on the expression of CAFs on the progression of GC. The results of the current study were in line with our hypothesis. In addition, univariate and multivariate regression analysis indicated that CXCL12 in CAFs was a variable to predict the prognosis of patients with gastric carcinoma. This was confirmed in *in vitro* studies using SGC7901 and BGC823 cells.

Our bioinformatics analysis was performed using transcriptome data of GC tissue and scRNA sequencing data of GC tissue. Compared with some previous studies, we used the analysis of scRNA data sets to analyze CXCLs and CXCRs in gastric cancer with more details. And we found the relationship between the expression of CXCL12-CXCR4 and the malignant progression of gastric cancer in CAFs. And verification of cytology and clinical case data also confirmed the data obtained in our previous study 30. A study by Fang et al. focused on the role of exosomes secreted by tumors on fibroblasts, which in turn affected the progression of tumors 31, and we believe that induced the secretion of proteins by tumor cells and impacted CAFs. This effect was more direct and more in line with the characteristics of gastrointestinal tumors. In the research on the malignant progress of GC cells, other teams have given us great inspiration for the research results of GC cell EMT. Overall, the novelty of our research lies in our integration of bioinformatics analysis, cell biology and clinical pathology evidence, and we proposed the mechanism of GC cell-CXCL12-CAFs-EMT in gastric cancer.

This study had some limitations. First, the data of our integrated bioinformatics analysis were derived from the GEO dataset. Although these datasets are derived from high-quality sequencing, due to the lack of our own omics samples, there were some restrictions throughout the research process. Second, the IHC tests were semi-quantitative and strongly dependent on the pathologist and the quality of antibodies. In addition, due to the limited lifespan of primary CAFs *in vitro*, human fibroblast cell lines were used for co-culture experiments.

## Conclusion

CXCL12 is frequently upregulated in CAFs of GC, and the combination of high CXCL12^CAF^ and Wnt5a is indicative of a poor prognosis of GC patients.

## Supplementary Material

Supplementary figures and tables.

## Figures and Tables

**Table 1:** Correlation between CXCL12 expression in cancer-associated fibroblast, tumoral Wnt5a and clinicopathological variables in 285 gastric cancer patients

Clinical parameters	CXCL12^CAF^		P value	Wnt5a		P value
	Positive (N=136)	Negative (N=149)		Positive (N=124)	Negative (N=161)	
**Gender**			0.903			0.231
Male	74 (54.4%)	80 (53.7%)		72 (58.1%)	82 (50.9%)	
Female	62 (45.6%)	69 (46.3%)		52 (41.9%)	79 (49.1%)	
**Age**			0.456			0.238
<60	57 (41.9%)	56 (37.6%)		54 (43.5%)	59 (36.6%)	
≥60	79 (58.1%)	93 (62.4%)		70 (56.5%)	102(63.4%)	
**Tumor size**			0.495			0.343
<3 cm	53 (39.0%)	64 (43.0%)		47 (37.9%)	70 (43.5%)	
≥3 cm	83 (61.0%)	85 (57.0%)		77 (62.1%)	91 (56.5%)	
**Histological grade**			0.012*			0.242
Well/moderately	51 (37.5%)	78 (52.3%)		61 (49.2%)	68 (42.2%)	
Poorly	85 (62.5%)	71 (47.7%)		63 (50.8%)	93 (57.8%)	
**Primary tumor category (T)**			0.286			0.016*
T1/T2	67 (49.3%)	64 (43.0%)		67 (54.0%)	64 (39.8%)	
T3/T4	69 (50.7%)	85 (57.0%)		57 (46.0%)	97 (60.2%)	
**Lauren type**			0.204			0.776
Intestinal	94 (69.1%)	113(75.8%)		89 (71.8%)	118(73.3%)	
Diffuse/Mix	42 (30.9%)	36 (24.2%)		35 (28.2%)	43 (26.7%)	
**TNM stage**			0.014*			0.147
I II	107 (78.7%)	133(89.3%)		100(80.6%)	140(87.0%)	
III IV	29 (21.3%)	16 (10.7%)		24 (19.4%)	21 (13.0%)	

Note: Values are n (%). *Significantly different by the Chi-squared test or Fisher exact probability method.

**Table 2:** Univariate and multivariate analysis of clinicopathological variables for overall survival rate in 285 gastric cancer patients

Clinical parameters	Univariate analysis	P value	Multivariate analysis	p value
	HR (95%CI)		HR (95%CI)	
Gender (female vs. male)	0.93 (0.73-1.20)	0.780		
Age (≥60 vs. <60)	0.84 (0.65-1.08)	0.183		
Tumor size (≥3 cm vs. <3 cm)	1.29 (1.00-1.66)	**0.047**	1.06 (0.82-1.38)	0.649
Histological grade (poor vs. well to mod.)	1.19 (0.92-1.53)	0.179		
Primary tumor category (T1/T2 vs. T3/T4)	0.55 (0.43-0.70)	**<0.001**	0.55 (0.42-0.72)	**<0.001**
Lauren type (diffuse/mix vs. intestinal)	1.58 (1.15-2.32)	**0.001**	1.64 (1.23-2.17)	**0.001**
TNM stage (III-IV vs. I-II)	1.13 (1.02-1.34)	**0.046**	0.98 (0.69-1.38)	0.894
CXCL12 ^CAF^ (positive/negative)	1.38 (1.07-1.76)	0.012*	1.50 (1.16-1.93)	0.002*
Wnt5a (positive/negative)	1.10 (1.41-1.81)	0.007*	1.28 (0.99-1.66)	0.060*

*Statistically significantly.

## References

[1] Abdelfatah MM (2018). Abdelfatah MM, Barakat M, Lee H, Kim JJ, Uedo N, Grimm I, Othman MO. The incidence of lymph node metastasis in early gastric cancer according to the expanded criteria in comparison with the absolute criteria of the Japanese Gastric Cancer Association: a systematic review of the literature and meta-analysis. Gastrointest Endosc.

[2] Jun N, Hisashi I, Hironori Y (2018). Clinical Importance of Epstein⁻Barr Virus-associated Gastric Cancer. Cancers (Basel).

[3] Choi Y K, Ahn J Y, Kim D H (2018). Efficacy and safety of endoscopic submucosal dissection for gastric neoplasms in patients with compensated liver cirrhosis: a propensity score-matched case-control study. Gastrointestinal Endoscopy.

[4] Karakasheva T A, Lin Eric W, Tang Qiaosi (2018). IL-6 Mediates Cross-Talk between Tumor Cells and Activated Fibroblasts in the Tumor Microenvironment. Cancer Res.

[5] Yasumoto K, Koizumi K, Kawashima A (2006). Role of the CXCL12/CXCR4 axis in peritoneal carcinomatosis of gastric cancer. Cancer Res.

[6] Cheng WL, Wang CS, Huang YH (2011). Overexpression of CXCL1 and its receptor CXCR2 promote tumor invasion in gastric cancer. Ann Oncol.

[7] Gosuke T, Michiru N, Kana K (2016). Wnt5a-Ror2 signaling in mesenchymal stem cells promotes proliferation of gastric cancer cells by activating CXCL16-CXCR6 axis. Cancer Sci.

[8] Chen Z, Tang C, Zhu Y (2017). TrpC5 regulates differentiation through the Ca2+/Wnt5a signalling pathway in colorectal cancer. Clin Sci (Lond).

[9] Cao B, Wang Q, Zhang H (2017). Two immune-enhanced molecular subtypes differ in inflammation, checkpoint signaling and outcome of advanced head and neck squamous cell carcinoma. Oncoimmunology.

[10] Iskender B, Izgi K, Hizar E (2016). Inhibition of epithelial-mesenchymal transition in bladder cancer cells via modulation of mTOR signalling. Tumour Biol.

[11] Pan B, Kusko R, Xiao W (2017). Dynamic characterization of drug resistance and heterogeneity of the gastric cancer cell BGC823 using single-cell Raman spectroscopy. Analyst.

[12] Lee K W, Yeo S Y, Sung C O (2015). Twist1 is a key regulator of cancer-associated fibroblasts. Cancer Res.

[13] Shen S, Chen X, Li H, Sun L (2018). MLH1 Promoter Methylation and Prediction/Prognosis of Gastric Cancer: A Systematic Review and Meta and Bioinformatic Analysis. J Cancer.

[14] Wang S, Zhang X, Li Z (2019). Circular RNA profile identifies circOSBPL10 as an oncogenic factor and prognostic marker in gastric cancer. Oncogene.

[15] Zhang P, Yang M, Zhang Y (2019). Dissecting the Single-Cell Transcriptome Network Underlying Gastric Premalignant Lesions and Early Gastric Cancer. Cell Rep.

[16] Taiwen Li, Jingyu Fan, Binbin Wang (2017). TIMER: A web server for comprehensive analysis of tumor-infiltrating immune cells. Cancer Research.

[17] Wang F, Wu J, Wang Y (2019). Gut Microbiota Functional Biomolecules with Immune-Lipid Metabolism for a Prognostic Compound Score in Epstein-Barr Virus-Associated Gastric Adenocarcinoma: A Pilot Study. Clin Transl Gastroenterol.

[18] Zhang C, Wang T, Wu H (2019). HEF1 regulates differentiation through the Wnt5a/β-catenin signaling pathway in human gastric cancer. Biochem Biophys Res Commun.

[19] Naijun Y, Guijuan Z, Fengjie B (2017). Integrative analysis of lncRNAs and miRNAs with coding RNAs associated with ceRNA crosstalk network in triple negative breast cancer. Onco Targets Ther.

[20] Teicher BA, Fricker SP (2010). CXCL12 (SDF-1)/CXCR4 pathway in cancer. Clin Cancer Res.

[21] Sun Y X, JinGCaheng Wang, Charles E (2003). Shelburne, et al. Expression of CXCR4 and CXCL12 (SDF-1) in human prostate cancers (PCa) *in vivo*. J Cell Biochem.

[22] Chinni S R, Sivalogan S, Dong Z (2006). CXCL12/CXCR4 signaling activates Akt-1 and MMP-9 expression in prostate cancer cells: the role of bone microenvironment-associated CXCL12. Prostate.

[23] Miao L (2017). Transient and Local Expression of Chemokine and Immune Checkpoint Traps To Treat Pancreatic Cancer. ACS Nano.

[24] Dickinson S C, Sutton C A, Brady K (2017). The Wnt5a Receptor, Receptor Tyrosine Kinase-Like Orphan Receptor 2, Is a Predictive Cell Surface Marker of Human Mesenchymal Stem Cells with an Enhanced Capacity for Chondrogenic Differentiation. Stem Cells.

[25] Park JY, Park KH, Bang S (2007). CXCL5 overexpression is associated with late stage gastric cancer. J Cancer Res Clin Oncol.

[26] Lee K, Hwang H, Nam K T (2014). Immune response and the tumor microenvironment: how they communicate to regulate gastric cancer. Gut Liver.

[27] Kanzawa M, Semba S, Hara S (2013). WNT5A is a key regulator of the epithelial-mesenchymal transition and cancer stem cell properties in human gastric carcinoma cells. Pathobiology.

[28] Ishimoto T, Miyake K, Nandi T (2017). Activation of Transforming Growth Factor Beta 1 Signaling in Gastric Cancer-associated Fibroblasts Increases Their Motility, via Expression of Rhomboid 5 Homolog 2, and Ability to Induce Invasiveness of Gastric Cancer Cells. Gastroenterology.

[29] Ma Y, Zhu Jing, Chen Shanwen (2018). Activated gastric cancer-associated fibroblasts contribute to the malignant phenotype and 5-FU resistance via paracrine action in gastric cancer. Cancer Cell Int.

[30] Li Q, Yang Y, Jiang X (2019). The combined expressions of B7H4 and ACOT4 in cancer-associated fibroblasts are related to poor prognosis in patients with gastric carcinoma. Int J Clin Exp Pathol.

[31] Fang T, Lv H, Lv G (2018). Tumor-derived exosomal miR-1247-3p induces cancer-associated fibroblast activation to foster lung metastasis of liver cancer. Nat Commun.

