# Supplementary material for: Cancer-associated fibroblasts in gastric cancer affect malignant progression via the CXCL12-CXCR4 axis
Source: J Cancer. 2021 Mar 19;12(10):3011–23. doi: 10.7150/jca.49707 (PMC8040897; doi:10.7150/jca.49707)
Supplement: Supplementary file 1 — Supplementary figures and tables. [file jcav12p3011s1.pdf]

supplementary figures

Figure S1

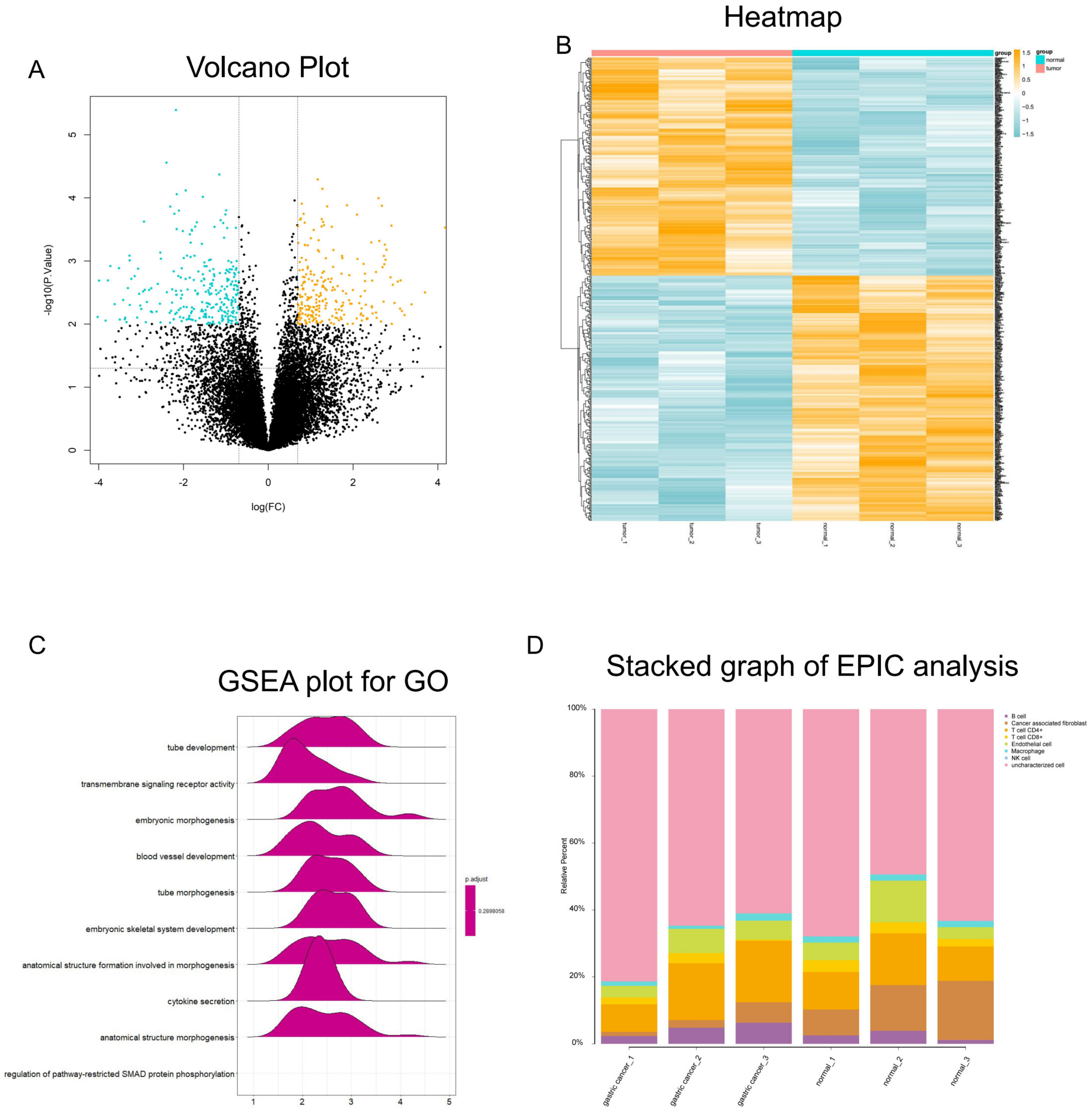

Figure S2

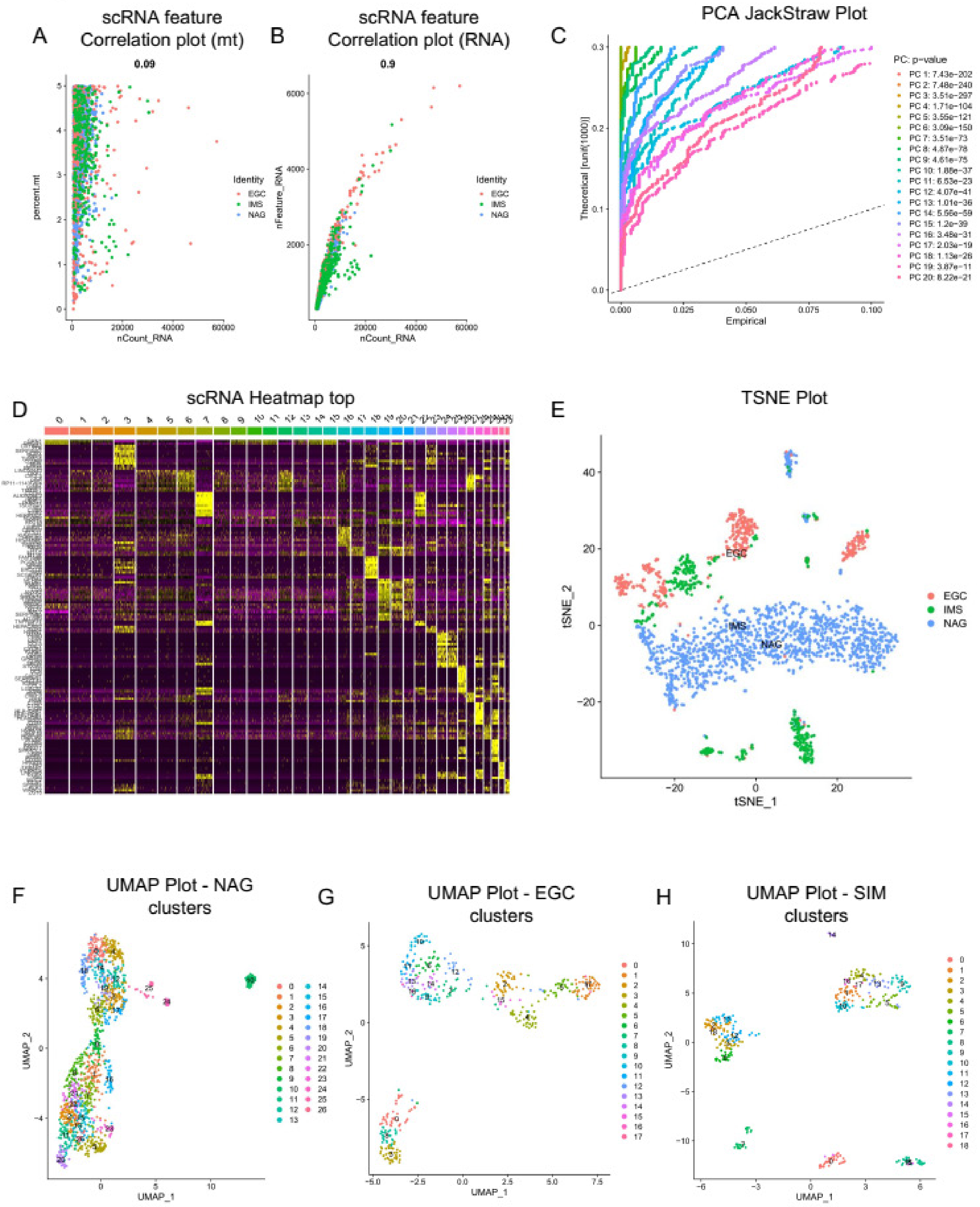
A
scRNA feature Correlation plot (mt)
0.09
percent.mt
nCount_RNA
Identity
EGC
IMS
NAG
B
scRNA feature Correlation plot (RNA)
0.9
nFeature_RNA
nCount_RNA
Identity
EGC
IMS
NAG
C
PCA JackStraw Plot
Theoretical [runif(1000)]
Empirical
PC: p-value
PC 1: 7.43e-202
PC 2: 7.48e-240
PC 3: 3.51e-297
PC 4: 1.71e-104
PC 5: 3.55e-121
PC 6: 3.09e-150
PC 7: 3.51e-73
PC 8: 4.87e-78
PC 9: 4.61e-75
PC 10: 1.88e-37
PC 11: 6.53e-23
PC 12: 4.07e-41
PC 13: 1.01e-36
PC 14: 5.56e-59
PC 15: 1.2e-39
PC 16: 3.48e-31
PC 17: 2.03e-19
PC 18: 1.13e-26
PC 19: 3.87e-11
PC 20: 8.22e-21
D
scRNA Heatmap top
E
TSNE Plot
tSNE_2
tSNE_1
EGC
IMS
NAG
F
UMAP Plot - NAG clusters
UMAP_2
UMAP_1
G
UMAP Plot - EGC clusters
UMAP_2
UMAP_1
H
UMAP Plot - SIM clusters
UMAP_2
UMAP_1

Figure S3

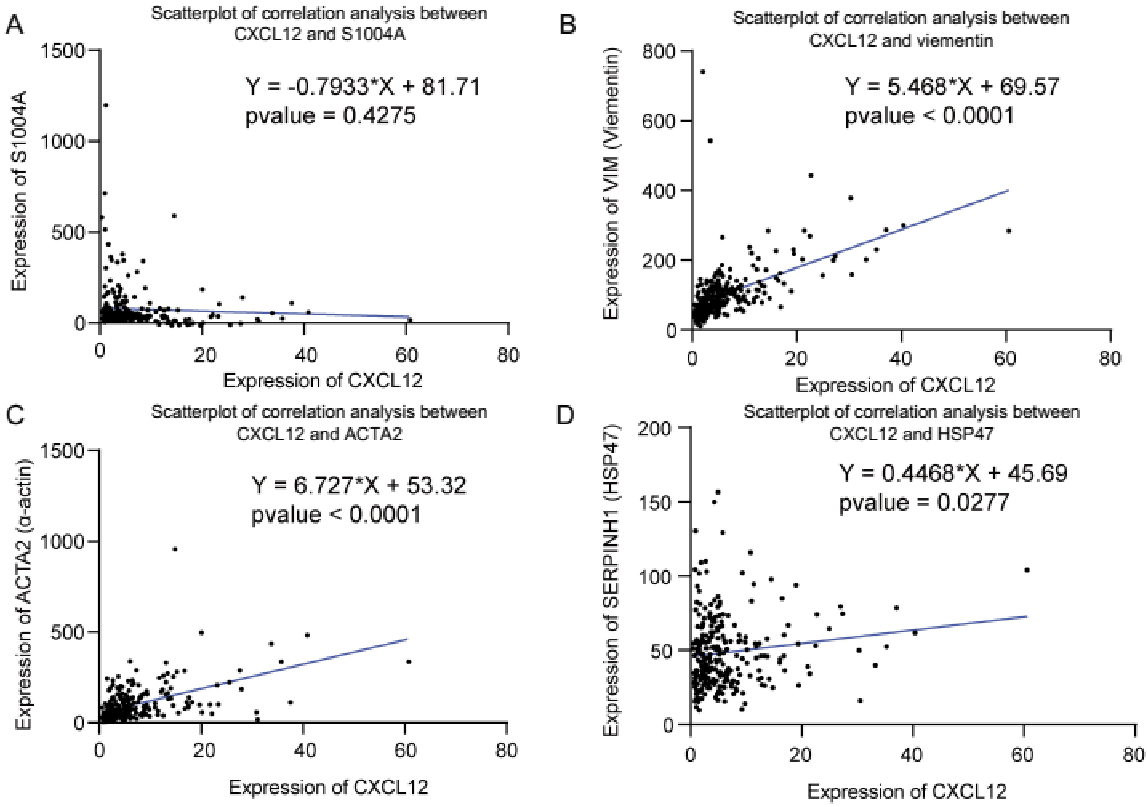
A
Scatterplot of correlation analysis between
CXCL12 and S1004A
Y = -0.7933*X + 81.71
pvalue = 0.4275
Expression of S1004A
Expression of CXCL12
1500
1000
500
0
0
20
40
60
80
B
Scatterplot of correlation analysis between
CXCL12 and viementin
Y = 5.468*X + 69.57
pvalue < 0.0001
Expression of VIM (Viementin)
Expression of CXCL12
800
600
400
200
0
0
20
40
60
80
C
Scatterplot of correlation analysis between
CXCL12 and ACTA2
Y = 6.727*X + 53.32
pvalue < 0.0001
Expression of ACTA2 (α-actin)
Expression of CXCL12
1500
1000
500
0
0
20
40
60
80
D
Scatterplot of correlation analysis between
CXCL12 and HSP47
Y = 0.4468*X + 45.69
pvalue = 0.0277
Expression of SERPINH1 (HSP47)
Expression of CXCL12
200
150
100
50
0
0
20
40
60
80

supplementary tables

Supplementary Table.1 Youden test of CXCL12^{CAF}

score	Sensitivity	1-specificity	Yoden index
2.5	0.532	0.36	0.172
5	0.266	0.099	0.167
1.5	0.718	0.553	0.165
3.5	0.371	0.267	0.104
7	0.113	0.043	0.07
0.5	0.815	0.752	0.063
8.5	0.073	0.012	0.061
10.5	0.016	0.012	0.004
-1	1	1	0
13	0	0	0

Supplementary Table.2 Youden test of Wnt5a

score	Sensitivity	1-specificity	Yoden index
2.5	0.532	0.36	0.172
3.5	0.427	0.273	0.154
1.5	0.637	0.571	0.066
5	0.21	0.161	0.049
0.5	0.653	0.627	0.026
8.5	0.056	0.043	0.013
7	0.073	0.062	0.011
10.5	0.024	0.019	0.005
-1	1	1	0
13	0	0	0

Supplementary Table.3 Logostics of CXCL12CAF, Primary tumor category, and Lauren type for 5-year survival rate of patients with gastric cancer.

Clinical parameters	Logistics analysis		P value
	β	OR(95%CI)	
	-0.268		0.382
Histological grade (poor vs. Well to mod)	-1.459	0.23(0.14-0.39)	<0.001
Lauren type (Diffuse/Mixvs.Intestinal)	1.100	3.01(1.67-5.43)	<0.001
CXCL12 ^{CAF} (positive/negative)	0.848	2.33(1.38-3.94)	0.002
